# A Three-Year Plant Study of Salt-Tolerant Transgenic Maize Showed No Effects on Soil Enzyme Activity and Nematode Community

**DOI:** 10.3390/life12030412

**Published:** 2022-03-11

**Authors:** Xing Zeng, Tongtong Pei, Yongfeng Song, Pei Guo, Huilan Zhang, Xin Li, Hao Li, Hong Di, Zhenhua Wang

**Affiliations:** Key Laboratory of Germplasm Enhancement, Physiology and Ecology of Food Crops in Cold Region, Department of Agriculture, Northeast Agricultural University, No. 600 Changjiang Street, Xiangfang District, Harbin 150030, China; zengxing@neau.edu.cn (X.Z.); ptt2387855149@163.com (T.P.); song18182746326@163.com (Y.S.); 18847163925@163.com (P.G.); z1025231075@163.com (H.Z.); lixin11170104@163.com (X.L.); lh_works@163.com (H.L.)

**Keywords:** salt-tolerant, transgenic maize, rhizosphere soil, enzyme activity, nematodes

## Abstract

The environmental effects of genetically modified crops are now a global concern. It is important to monitor the potential environmental impact of transgenic corn after commercial release. In rhizosphere soil, plant roots interact with soil enzymes and microfauna, which can be affected by the transgenes of genetically modified crops. To determine the long-term impact of transgenic plant cultivation, we conducted a field study for 3 consecutive years (2018–2020) and observed the enzyme activities and nematode populations in plots planted with transgenic maize BQ-2, non-transgenic wild-type maize (Qi319), and inbred line B73. We took soil samples from three cornfields at four different growth stages (V3, V9, R1, and R6 stages); determined soil dehydrogenase, urease, and sucrase activities; and collected and identified soil nematodes to the genus level. The results demonstrated seasonal variations in dehydrogenase, urease, and sucrase activities. However, there was a consistent trend of change. The generic composition and diversity indices of the soil nematodes did not significantly differ, although significant seasonal variation was found in the individual densities of the principal trophic groups and the diversity indices of the nematodes in all three cornfields. The results of the study suggest that a 3-year cultivation of transgenic corn had no significant effects on soil enzyme activity and the soil nematode community. This study provides a theoretical basis for the environmental impact monitoring of transgenic corn.

## 1. Introduction

The salinization of soils is one of the more severe environmental problems associated with crop productivity in China and worldwide, and global warming-induced increases in sea levels will likely intensify this problem. Therefore, the most viable strategy for increasing crop production is to develop more salt-tolerant varieties of plants, especially the staple crop maize (*Zea mays* L.).

Transgenic breeding is a new breeding method with the characteristics of short cycles and high efficiency, and it has become the most important technology in the field of crop breeding. According to ISAAA, from 1996 to 2019, the global GM crop planting area reached 190.4 million hm^2^, an increase of more than 100 times [1]. With the increase of population, the area of arable land continues to decrease. To improve the utilization rate of arable land and increase the yield of corn, an urgent global need has arisen to cultivate genetically modified varieties with insect resistance, herbicide resistance, drought resistance, salinity resistance, and high quality through biotechnology.

With the commercial cultivation of genetically modified organisms, there has been increasing concern about risks to the environment, including the impact on the soil ecosystem [2]. Exogenous genes (such as, Bt insecticidal crystal protein genes, protease inhibitor genes, antibiotic genes, etc.) in transgenic crops can remain in the soil through root exudates, pollen, and residual branches and leaves, which may directly or indirectly affect soil nutrients, physicochemical properties, enzyme activities, soil animals, and microorganisms [3]. For example, when the Bacillus thuringiensis (Bt) toxin is released into the soil, it is adsorbed or bound on humus, mineral–organic complexes, or clay particles [4]. Soil enzymes and soil microorganisms are important components of soil and are indispensable members of the material cycle. They are mainly involved in the accumulation of soil organic matter and mineralization processes, affecting soil nutrient cycling and soil nutrient availability, and play an important role in plant growth. Genetically modified crops can affect the physiology, metabolism, community composition, and soil enzyme activities of soil microorganisms through their residues, thereby affecting the physicochemical properties and nutrient availability of soil. Dong et al. found that the pH of the rhizosphere soil of transgenic cotton increased [5]. The study by Wang et al. showed that the effect of chitinase transgenic tobacco on soil pH varied with different plant growth stages [6]. Other studies have shown that the transgenic *BADH* soybean has a certain degree of influence on the availability of soil phosphorus content, but that the difference is temporal [7]. The activities of urease, nitrate reductase, acid phosphatase, and alkaline phosphatase were higher in soil cultivated with Bt transgenic cotton [8]. However, transgenic cotton had no significant effect on the activities of phenoloxidase, dehydrogenase, phosphatase, protease, and urease in rhizosphere soil [9].

On the other hand, soil is the hub of material and energy exchange in agricultural ecosystems, and soil organisms have special functions and roles in the process of soil material and energy migration and transformation. Preliminary investigations have shown that Bt cotton exhibits no significant negative effects on soil fauna and flora, and may even be beneficial to these communities [10,11]. Soil nematodes are widely distributed in soil. They feed on bacteria, fungi, other nematodes, and plant tissues in the soil. They have important functions, such as decomposing organic matter and nutrients and maintaining the ecological balance of soil. They are extremely sensitive to changes in the soil’s ecological environment. As an important member of the soil ecosystem, soil nematodes have become indicator organisms that indicate changes in the soil’s ecological environment [8]. One important potential risk is the negative impact of genetically modified crops on soil non-target organisms. Studies on the effects of genetically modified crops on non-target organism soil nematodes have been extensively reported, albeit with mixed results. Most research results show that transgenic crops do not affect soil nematode diversity [12,13,14]. The populations and/or numbers of soil organisms (e.g., nematode fungi and bacteria) were not affected, and the expression of *SUV3* or *PDH45* in rice might not lead to changes in soil enzyme activities, functional diversity, or microbial communities in rhizosphere soils [15,16]. However, some studies have pointed out that the planting of transgenic crops will affect the individual indicators and species of soil nematodes [17,18,19]. Previous studies on the effects of transgenic crops on soil nematode communities have mostly focused on transgenic Bt-resistant crops. There have been relatively few studies on the effects of salinity-tolerant, drought-tolerant, and other reversal-tolerant crops on soil nematode communities.

However, the soil environment is a very complex ecosystem, in which many factors affect soil biota. In field studies, a high variability in biotic parameters is inherent and usually present, and this variability must be considered seriously if the ecological risks posed by transgenic plants are to be monitored. Moreover, soil biota may be strongly stressed as a result of the influence of environmental factors (e.g., pH, salinity, redox potential, vegetation, and water-holding capacity), which may cause higher or lower levels of sensitivity to transgenic plants [15,18,19]. In addition, according to the current research, most associated studies have been conducted under laboratory and semi-field conditions, and most studies have examined the possible effects of cultivating insect-resistant or herbicide-tolerant transgenic plants. Moreover, these studies were conducted in fields with short histories of transgenic cultivation, within a short period. In our preliminary research, we obtained a transgenic line BQ-2, which introduced the betaine aldehyde dehydrogenase (*BADH*) gene into the maize inbred line Qi319, then generated a strong, salt-tolerant phenotype, even at a high concentration of NaCl [20]. Therefore, to evaluate the safety of the anti-retroversion maize gene to the soil ecological environment of maize fields, the transgenic maize line BQ-2, receptor inbred line Qi319, and maize inbred line B73 were employed as our experimental treatments. The potential effects of transgenic plants on the activities of dehydrogenase, urease, and sucrase, as well as the soil nematode community in rhizosphere soils, were further evaluated to provide environmental safety data for the commercial application of transgenic stress-tolerant maize.

## 2. Materials and Methods

### 2.1. Plant Materials and Field Trial

Three maize species, including the transgenic maize BQ-2, the wild-type maize Qi319, and the maize inbred line B73, were used in this study. The three materials were grown in saline soil in triplicate, from 2018 to 2020, at the Transgenic Experiment Station of Northeast Agricultural University, Harbin, Heilongjiang, China (longitude 126°73′, latitude 45°75′). The experimental site is located in the temperate zone and has a continental monsoon climate. The fields had a saline-type soil (FAO (1998) classification) with a soil–water ratio of 1:2.5, pH of 8.65, total N of 127.27 mg·kg^−1^, total P of 170.47 mg·kg^−1^, total K of 385.01 mg·kg^−1^, organic C of 27.08 g·kg^−1^, available P of 23.54 mg·kg^−1^, and available K of 178.91 mg·kg^−1^ on a dry mass basis.

The BQ-2, Qi319, and B73 plants were distributed side by side with 2-m wide belts separating the fields. The annual maize growing season was from May to October. The agricultural practices for the maize in all fields were identical to those used in standard corn production, with no chemical pesticides throughout the growing season due to pest abundance and poor crop health. The manual removal of weeds occurred on a routine basis. After harvesting, the whole plant was cut and returned to the soil as an organic fertilizer. All fields were allowed to lay fallow from November until April the following year.

### 2.2. Soil Sample Collection

From 2018 to 2020, three independent samples of rhizosphere soils from each plot were sampled at the V3, V9, R1, and R6 stages of maize plant growth. To avoid margin effects, the five meters at both ends of each field were not sampled. All fields were divided into 3 subplots. After removing the leaves and weeds, the surface soils (*n* = 3) were collected from each subplot by following the checkerboard method [21]. Briefly, the soils were extracted from a depth between 0 and 20 cm using a soil auger (diameter = 4 cm) and then placed in a sterile plastic bag. All samples were immediately returned to the laboratory and stored in the dark at 4 °C until further analysis. The data of soil samples (*n* = 3) in each subplot were pooled, and the average values from the three replicates were subjected to statistical analysis.

### 2.3. Soil Enzyme Assays

The soil urease activities were evaluated using the ‘the loss of added urea’ method [22]. A total of 5 g of dry soil was carefully transferred into a 25 mL measuring flask, and 1 mL of toluene was added. After standing for 15 min, 10 mL of 10% urea solution and 20 mL of citric acid buffer (pH 6.7) were incorporated. The soil sample and the added solutions were mixed evenly and incubated at 37 °C for 24 h, followed by filtering. Then, 3 mL of the filtrate was mixed with 20 mL of distilled water, 4 mL of 12.5% (*w*/*v*) potassium sodium tartrate, and 3 mL of hypochlorous acid sodium solution (0.9% active chlorine). The color was allowed to develop for 20 min; the solution was then diluted to 50 mL and the absorbance of the diluted solution was measured at 578 nm in an ultraviolet spectrometer subsystem (UVS) (PGENARAL T6). Soil urease activities are expressed as milligrams of NH^4+^-N per gram of soil per 24 h period.

Sucrase activities were detected using a 3,5-dinitrosalicylic acid reagent [23]. Then, 2 g of fresh soil, 15 mL of 8% glucose solution, 5 mL of 0.2 M phosphate buffer (pH 5.5), and 0.25 mL toluene were mixed in a 50 mL volumetric flask and incubated for 24 h at 37 °C. The mixture was filtered, a 1 mL filtrate was drawn, then reacted with 3 mL of 3,5-dinitrylsalicylate for 5 min in boiling water; the products were then quantified using a spectrophotometer at 508 nm. Soil sucrase activities are expressed as milligrams of glucose per gram per 24 h period.

Dehydrogenase activities were measured by following a previously used method [24]; 5 g of fresh soil was incubated for 24 h at 37 °C in 5 mL of a TTC solution (5 g TTC in 0.2 mol/L Tris HCl buffer, pH 7.4). Two drops of concentrated sulfuric acid were added immediately after the incubation to end the reaction. The sample was then blended with 20 mL of methanol and shaken for 1 h at 200 rpm, followed by filtering to extract TPF. The products were then quantified using a spectrophotometer at 485 nm. Soil dehydrogenase activity is expressed as the micrograms of TPF per gram of soil per 24 h period.

### 2.4. Enumeration of Soil Nematode Populations

Nematodes were extracted from the rhizosphere soils of BQ-2, Qi319, and B73 using the Baermann method [24,25]. Nematode specimens from the soil samples were uniformly distributed over tissue paper in a water suspension supported by a screen on a Petri dish, which was filled with water. After the samples were set, the Petri dishes were covered with caps and incubated at 23–25 °C for 36 h. To calculate the total number of nematodes, the content in the Petri dishes was transferred to a flat-bottomed, partitioned counting dish (36 squares). A Nikon Stereoscopic Zoom Microscope (10–100×) was used to examine the number of nematodes in 10 random squares. The total number of nematodes in each sample was determined by multiplying the average count in 10 squares by 36.

### 2.5. Experimental Design and Statistical Analyses

The identified nematodes were assigned to bacterivores (Ba), fungivores (Fu), omnivore–predators (OP), and plant parasites (PP) [26,27]. Then, the following nematode community indices were calculated [28,29]:

(1) Dominant genera (relative abundance  >  10%, dominant genera; 1%  <  relative abundance  <  10%, common genera; relative abundance  <  1%, rare genera);
(2) Species richness index (S);
(3) Shannon–Wiener diversity index (H), $H=-\sum_{i=1}P_{i}{\mathrm{lnP}}_{i}$;
(4) Dominance index (C), $C=\sum_{i=1}{\left(P_{i}\right)}^{2}$;
(5) Pielou’s evenness index (J), J =$\;\frac{H}{\ln S}$;
(6) Nematode channel ratio (R_NC_), R_NC_ =$\;\frac{B}{B+F}$;
(7) Wasilewska index (I_W_), I_W_ =$\;\frac{B+F}{P}$;
(8) Maturity index (I_M_), I_M_ = ∑v(i)f(i);
(9) Phytophagous nematode index (I_PP_).

In the above equations, S is the total number of nematode genera in the community; Pi is the proportion of the individuals of the ith group in the community; v(i) is the c-*p* value of the ith taxon; f(i) is the frequency of the ith taxon; and B/F/P are the numbers of bacterivores/fungivores/plant parasites in the total soil nematode population.

All nematode counts were converted to 100 g dry soil, and the relative abundance and dominance of each nematode genus were calculated. All data were processed using Excel 2010 and SPSS 16.0 (SPSS Inc., Chicago, IL, USA), and a variance analysis was performed on each index by univariate analysis. Data are expressed as the mean ± standard deviation (SD).

## 3. Results

### 3.1. Activities of the Three Enzymes in Rhizosphere Soil

**Figure 1:**
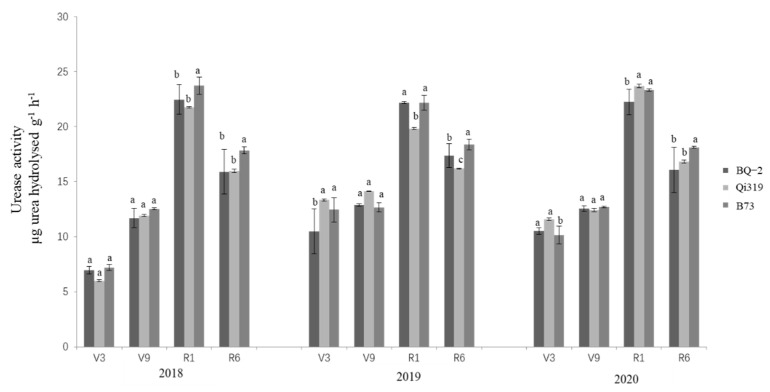
**Urease activity in three cornfields at different sampling times.** Note: the different letters above the bars denote a statistically significant difference between the means of the fields. V3, the three lowest leaves with a visible collar; V9, nine leaves with collars; R1, silking; R6, physiological maturity.

**Figure 2:**
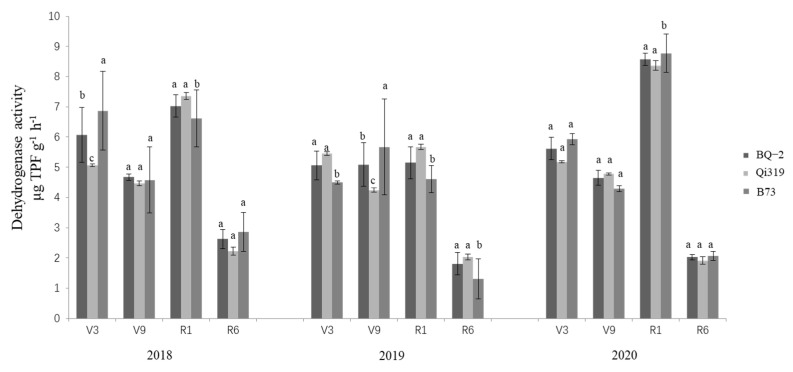
**Dehydrogenase activity in three cornfields at different sampling times.** Note: the different letters above the bars denote a statistically significant difference between the means of the fields. V3, the three lowest leaves with visible collar; V9, nine leaves with collars; R1, silking; R6, physiological maturity.

**Figure 3:**
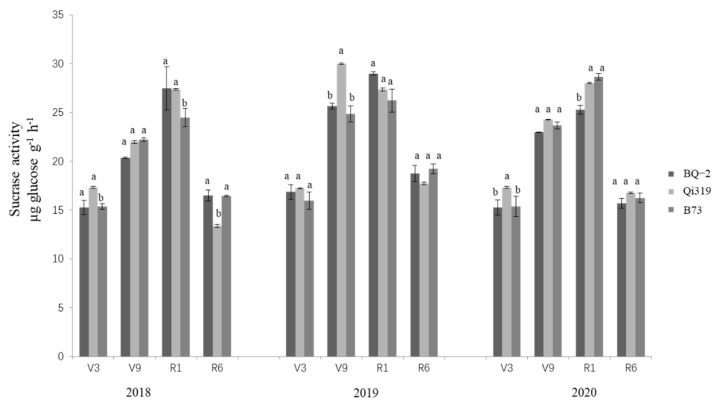
**Sucrase activity in three cornfields at different sampling times.** Note: the different letters above the bars denote a statistically significant difference between the means of the fields. V3, the three lowest leaves with a visible collar; V9, nine leaves with collars; R1, silking; R6, physiological maturity.

The effects of transgenic BQ-2 on urease, dehydrogenase, and sucrase activity compared with recipient inbred line Qi319 and another maize inbred line B73 in saline–alkaline soil are displayed in Figure 1, Figure 2 and Figure 3. During the 3-year period (2018–2020) of the study, the main factor affecting the activities of urease, dehydrogenase, and sucrase was sampling time, regardless of the planting material (Table 1). During the period of crop growth, the activities of urease, dehydrogenase, and sucrase were relatively lower in rhizosphere samples at the V3 vegetative stage, although the activities of the three enzymes were higher at the V9 and R1 stages. At the R6 stage of crop harvesting, the activities of all enzymes had declined. In general, the activities of urease and sucrase showed an increasing–decreasing trend, reaching a peak in the R1 stage; dehydrogenase activity increased slightly in the V3, V9, and R1 stages, and was significantly decreased in the R6 stage.

### 3.2. Abundance Changes of Soil Nematode Trophic Groups in the Three Maize Fields

From 2018 to 2020, through morphological identification, a total of 37 nematode taxa were found across the planting fields of three materials (Table 2), including 14 genera of bacterivores, 4 genera of fungivores, 9 genera of omnivores–predators, and 10 genera of plant parasites. The dominant genera, Acrobeles, Cephalobus, and Pratylenchus, were identified, accounting for 20.2%, 13.1%, and 18.9% of the total number of nematodes, respectively. Moreover, there were 15 genera of common taxa, accounting for 37.9% of the total, and 19 genera of rare taxa, accounting for 9.8% of the total. According to nematode functional groups, the most abundant feeding group was bacterial feeders, followed by plant parasites and omnivore–predator nematodes.

From 2018 to 2020, there was no significant difference in soil nematode abundance and phytophagous nematode abundance between saline–alkaline-tolerant transgenic maize BQ-2, receptor control Qi319, and common maize inbred line B73 (Table 2). Among them, the abundance of soil bacteria-eating nematodes of Bq-2 was between QI319 and B73, and the abundance of predatory omnivorous nematodes was significantly lower than that of QI319, but there was no statistical difference with the data of b73 (Table 3). The results of ANOVA also showed that the total number of soil nematodes and the nematodes of each nutritional type were not affected by the sampling time, and there was no interaction between the sampling time and traits (Table 4).

### 3.3. Changes of Nematode Community Indices in Different Corn Fields

A variance analysis showed that most soil nematode community-related indices were not different for plant BQ-2, control receptor Qi319, and maize inbred line B73 for three consecutive years, such as the species richness index (S), dominance index (C), Wasilewska index (I_W_), and phytophagy nematode index (I_PP_). However, we found that the diversity index (H), Pielou evenness index (J), and maturity index (I_M_) were slightly different (Table 5). A further analysis found that the difference in the H index and the J index mainly came from qi319; the difference in the IM index mainly came from the conventional inbred line B73; there was no difference between the transgenic material BQ-2 and the receptor Qi319; and there was no interaction between the sampling time and traits (Table 6).

## 4. Discussion

The *BADH* gene was responsible for the synthesis of glycine betaine in various plant species. A plant that inhabits saline and arid soils may accumulate glycine betaine due to high salt stress. Accumulation of glycine betaine has gained increasing interest in the field of plant salinity tolerance [30]. Previous studies have shown that plants with glycine betaine deficiency exhibit a low tolerance for heat and salinity [31,32,33]. A method for introducing the *BADH* gene into plants with a defect in the glycine betaine pathway that reduces their salt–stress resistance, such as Arabidopsis, Ammopiptanthus nanus, maize, potato, Persian walnut, wheat, etc., has been proposed [34,35,36,37,38,39]. With the continuous development of transgenic plant varieties, monitoring the environment after their large-scale cultivation has received increasing attention in the past 20 years, especially insect-resistant or herbicide-tolerant crops [40,41,42,43,44,45,46]. As a result, the potential non-target impacts caused by transgenic plants can form a critical component of risk assessments in environmental management.

In this study, we analyzed the role of salt-tolerant *BADH*-overexpression in rhizosphere soils, to evaluate the impact of long-term cultivation on the soil environment. Our findings indicate that introducing transgenic maize with the *BADH* gene may not affect the soil’s enzyme activity or nematode populations in rhizosphere soils. Although several reports demonstrated adverse impacts of transgenic crops, others did not observe any differences in the compositions of rhizosphere soils; most differences could be related to agricultural operations, climate, sampling time, etc. [47,48,49,50,51,52]. However, salinity-tolerant transgenic rice (*PDH45* and *SUV3* genes) or transgenic maize (*BcWRKY1* gene) might not induce significant alterations to soil enzyme activities, microbial communities, and functional diversity in rhizosphere soils [15,16,53]. Thus, our findings indicated no obvious differences in the urease activities, sucrase activities, and dehydrogenase activities of rhizosphere soils between transgenic and non-transgenic maize; this is in agreement with other reported findings; for example, in relation to the salinity tolerant *PDH45* transgenic rice, *SUV3* transgenic rice, and *BcWRKY1* transgenic maize, there were no significant differences in the activities of dehydrogenase, urease, nitrate reductase, and phosphatase between the transgenic and non-transgenic crop in rhizosphere soils [15,16,53].

Throughout our three-year field investigation, we found no significant difference in the total nematode community structure between BQ-12, the corresponding receptor control Qi319, and common inbred control B73. However, compared with the control field, the abundance of omnivorous nematodes in the transgenic field changed significantly, especially the abundance of Dorylaimus and Mesodorylaimus nematodes, which significantly decreased (*p* < 0.05). In the survey results, the significant differences between transgenic maize and its control fields only related to individual feeding habits and species of nematodes. It is speculated that the effect of transgenic maize on nematodes may be due to differences in feeding habits and species. Hoss found that the cultivation of transgenic maize (MON88017) had no effect on the number of soil nematodes in three consecutive years [54]; however, Griffiths demonstrated that the number of soil nematodes in the field of Bt transgenic maize was significantly reduced [12]. Bt rape (*Cry1Ac* gene) increases the proportion of fungal-eating nematodes and decreases the proportion of phytophagous nematodes [55]. Bt rice increases the proportion of herbivorous and predatory omnivorous nematodes [56]. The phytase gene transgenic maize did not cause changes in nematode community diversity and abundance, but the abundance and diversity indices of bacterial-eating nematodes increased, while the abundance and diversity indices of herbivorous nematodes decreased [18]. Recent reports indicate that Bt crop reduced phytoparasitic nematode abundance and enhanced trophic connections, but did not affect other nematode parameters in paddy fields or community composition [57,58]. It can be seen that some transgenic plants can, indeed, affect the abundance of different feeding nematodes. Therefore, when evaluating the ecological safety of transgenic plants, it would be worthwhile to perform further in-depth studies and select nematodes with sensitive feeding habits and species as evaluation indicators.

During the 3-year study period, there were individual changes in soil enzyme activities and the soil nematode population diversity index in all corn fields. However, compared with the recipient control Qi319 and the common maize inbred line B73, the long-term planting of BQ-2 did not affect the consistency of rhizosphere soil enzyme activities and the diversity of nematode populations. Conventional research suggests that soil enzymes play important roles in organic matter decomposition and nutrient cycling. Oxidoreductases and hydrolases are the two main categories of soil enzymes, and dehydrogenases, ureases, and sucrases belong to these; they are more sensitive to saline–alkali stress than other types of enzymes [16,17,59]. Therefore, they are used as important monitors of soil ecosystem quality and microbial community health [60,61,62]. This indicator is also used to evaluate the impact of genetically modified crops on soil. In this study, soil sucrase, urease, and dehydrogenase activities were used as evaluation objects, which is consistent with Bai’s study. Cropping BZ-136 had no effect on soil catalase and saccharase activity in natural or saline–alkaline soil, but there was a propitious effect on urease activities during the whole growth cycle in saline–alkaline soil [63]. On the other hand, although the number of individual nematode genera varied significantly in the timespan of this study, the overall community index was not affected by the sampling year. It is speculated that differences in community indices, such as the I_M_ index, come from these significantly different nematode genera. Therefore, future research should focus on considering these individual genera of nematodes as indicator organisms for evaluating the environmental safety of transgenic stress-tolerant maize.

Environmental risk assessments of insect resistant and herbicide tolerant crops over the past 10 years have shown that GM crop cultivation has had no impact on soil physicochemical properties, enzymatic activities, and soil biomes such as nematodes, earthworms, springtails, or mites [62,64,65,66,67,68,69,70,71]. However, the environmental safety assessment of stress-tolerant genetically modified crops, such as drought tolerance and salinity tolerance, was still insufficient. In this study, three years of data showed that transgenic salinity tolerance had no significant effect on soil enzyme activity and nematode community structure. It is necessary to continue monitoring the effects of transgenic plants on the soil ecosystem in different environments and to define the ecological significance of the planting of transgenic crops.

## 5. Conclusions

The experimental results showed that the continuous planting of transgenic saline–alkali-tolerant maize had no effect on urease, dehydrogenase, and sucrase in rhizosphere soil, compared with its receptor control and another common maize inbred line. The dominant groups of soil nematodes were the same, and there was no significant difference in the total number of nematodes, the abundance of different nematode trophic types, and the nematode community diversity index. Therefore, based on these findings, the effects of the *BADH* maize line (BQ-2) on rhizosphere soil physicochemical properties and nematode communities were within the expected variability.

## Figures and Tables

**Table 1:** ANOVA of enzyme activities in rhizosphere soils.

Source	df	F	P
Urease activity			
maize variety	2	0.66	0.42
Sampling time	11	51.40	0.00
Dehydrogenase activity			
maize variety	2	0.10	0.75
Sampling time	11	10.28	0.00
Sucrase activity			
maize variety	2	0.21	0.64
Sampling time	11	24.43	0.00

**Table 2:** Proportional contributions (%) of the various genera to the nematode assemblages from 2018 to 2020.

Genus	c-*p*	B73		BQ-2		Qi319	
		Relative Frequency (%)	Dominance	Relative Frequency (%)	Dominance	Relative Frequency (%)	Dominance
**Bacterivores**							
Acrobeles	2	20.27 ± 0.28 a	+++	20.05 ± 0.49 a	+++	20.35 ± 0.04 a	+++
Acrobeloides	2	2.94 ± 0.08 a	++	2.97 ± 0.03 a	++	2.71 ± 0.05 b	++
Alaimus	4	3.04 ± 0.03 b	++	3.43 ± 0.02 a	++	2.60 ± 0.06 c	++
Cephalobus	2	12.52 ± 0.26 c	+++	13.20 ± 0.31 b	+++	13.73 ± 0.07 a	+++
Cervidellus	2	1.33 ± 0.01 b	++	1.45 ± 0.03 a	++	1.35 ± 0.04 b	++
Chiloplacus	2	0.39 ± 0.02 b	+	0.45 ± 0.05 b	+	0.58 ± 0.03 a	+
Eucephalobus	2	0.75 ± 0.03 b	+	0.85 ± 0.07 a	+	0.78 ± 0.01 ab	+
Mesorhabditis	1	1.17 ± 0.05 a	++	1.00 ± 0.01 b	++	0.92 ± 0.01 c	++
Caenorhabditis	1	8.83 ± 0.36 a	++	7.35 ± 0.17 b	++	7.80 ± 0.13 b	++
Prismatolainus	3	0.73 ± 0.03 a	+	0.73 ± 0.04 a	+	0.76 ± 0.01 a	+
Protorhabditis	1	4.90 ± 0.10 b	++	4.90 ± 0.02 b	++	4.64 ± 0.03 a	++
Rhabditis	2	3.76 ± 0.47 a	++	3.96 ± 0.07 a	++	4.01 ± 0.06 a	++
Placodira	2	1.29 ± 0.05 a	++	1.21 ± 0.02 b	++	1.13 ± 0.01 c	++
Plectus	2	0.76 ± 0.02 ab	+	0.71 ± 0.02 b	+	0.81 ± 0.04 a	+
**Fungivores**							
Aphelenchus	2	1.31 ± 0.01 b	++	1.40 ± 0.01 a	++	1.32 ± 0.04 b	++
Ditylenchus	2	2.80 ± 0.02 a	++	2.78 ± 0.08 a	++	2.80 ± 0.11 a	++
Doryllium	4	0.38 ± 0.01 b	+	0.37 ± 0.02 b	+	0.55 ± 0.01 a	+
Tylencholaimus	4	0.50 ± 0.01 b	+	0.55 ± 0.05 ab	+	0.57 ± 0.02 a	+
**Omnivore–predators**							
Aporcelaimus	5	0.34 ± 0.03 b	+	0.28 ± 0.03 c	+	0.42 ± 0.03 a	+
Discolaimus	5	0.45 ± 0.01 a	+	0.39 ± 0.04 ab	+	0.38 ± 0.03 b	+
Dorylaimus	4	0.81 ± 0.03 b	+	0.93 ± 0.02 a	+	0.92 ± 0.03 a	+
Eudorylaimus	4	0.73 ± 0.04 a	+	0.79 ± 0.09 a	+	0.72 ± 0.01 a	+
Mesodorylaimus	5	0.37 ± 0.02 b	+	0.34 ± 0.04 b	+	0.68 ± 0.03 a	+
Microdorylaimus	4	0.44 ± 0.03 a	+	0.40 ± 0.04 a	+	0.42 ± 0.03 a	+
Doryllium	4	1.36 ± 0.03 a	++	1.37 ± 0.01 a	++	1.40 ± 0.04 a	++
Enchodelusthorne	4	0.38 ± 0.03 a	+	0.18 ± 0.04 b	+	0.35 ± 0.03 a	+
Thorneella	4	0.17 ± 0.01 a	+	0.12 ± 0.03 a	+	0.18 ± 0.05 a	+
**Plant parasites**							
Filenchus	2	1.06 ± 0.06 b	++	1.01 ± 0.05 b	++	1.21 ± 0.05 a	++
Helicotylenchus	3	1.90 ± 0.18 b	++	1.91 ± 0.23 b	++	2.29 ± 0.01 a	++
Nothotylenchus	2	1.32 ± 0.03 b	++	1.45 ± 0.08 a	++	1.26 ± 0.05 b	++
Pratylenchus	3	19.18 ± 0.21 a	+++	19.43 ± 0.22 a	+++	18.12 ± 0.53 b	+++
Psilenchus	3	0.27 ± 0.01 a	+	0.26 ± 0.06 a	+	0.29 ± 0.04 a	+
T’ylenchorhynchus	3	0.61 ± 0.01 c	+	0.73 ± 0.03 b	+	0.80 ± 0.01 a	+
Tylenchus	3	1.66 ± 0.03 a	++	1.72 ± 0.03 a	++	1.64 ± 0.06 a	++
Oxydirus	5	0.58 ± 0.01 a	+	0.54 ± 0.02 b	+	0.58 ± 0.03 ab	+
Cephalenchus	3	0.33 ± 0.03 b	+	0.33 ± 0.06 b	+	0.47 ± 0.03 a	+
Xiphinema	5	0.36 ± 0.02 b	+	0.45 ± 0.05 a	+	0.48 ± 0.03 a	+

Note: relative frequency (%) = (number of nematodes in a certain genus)/(observed number of nematodes in each habitat) × 100. +++: dominant genus, ++: common genus, +: rare genus. All values are given as mean + SD, different letters represent significant differences from each other at *p* < 0.05 level.

**Table 3:** Abundances of different trophic groups of soil nematodes in soils under transgenic maize, and the corresponding non-transgenic maize.

Material	Ba	Fu	OP	PP	Total
BQ-2	1176.22 ± 21.99 a	96.33 ± 0.67 ab	90.67 ± 3.28 b	526.11 ± 2.46 a	1889.33 ± 20.51 a
Qi319	1175.44 ± 2.83 a	99.22 ± 3.37 a	103.44 ± 3.66 a	513.22 ± 12.74 a	1891.33 ± 17.14 a
B73	1180.67 ± 11.86 a	94.11 ± 1.17 b	95.00 ± 1.86 b	513.89 ± 5.74 a	1883.67 ± 18.93 a

Note: Ba, bacterivores; Fu, fungivores; PP, plant parasite; OP, omnivore–predators. All values are given as mean + SD; different letters represent significant differences from each other at *p* < 0.05 level.

**Table 4:** Variance analysis of different nematode trophic groups.

Factor	Ba	Fu	OP	PP	Total
Material	0.14	1.22	7.03 **	1.80	0.11
years	2.35	0.50	1.39	1.17	2.54
Material × Year	0.62	0.16	0.07	0.56	0.01

Note: Ba, bacterivores; Fu, fungivores; PP, plant parasite; OP, omnivores–predators. ** Significant at *p*  <  0.01.

**Table 5:** Soil nematode community ecological indices of the soils under transgenic and non-transgenic corn.

Material	S	H	C	J	R_NC_	I_W_	I_M_	I_PP_
BQ-2	4.77 ± 0.01 a	2.71 ± 0.02 b	0.11 ± 0.00 a	0.75 ± 0.00 b	0.92 ± 0.00 b	2.42 ± 0.05 a	2.34 ± 0.01 a	0.83 ± 0.01 a
Qi319	4.77 ± 0.01 a	2.75 ± 0.01 a	0.11 ± 0.00 a	0.76 ± 0.00 a	0.92 ± 0.00 b	2.49 ± 0.05 a	2.34 ± 0.00 a	0.81 ± 0.01 a
B73	4.77 ± 0.01 a	2.71 ± 0.01 b	0.11 ± 0.00 a	0.75 ± 0.00 b	0.93 ± 0.00 a	2.48 ± 0.01 a	2.32 ± 0.00 b	0.81 ± 0.00 a

Note: S is species richness index, H is diversity index, C is dominance index, J is Pielou evenness index, RNC is nematode pathway index, IW is Wasilewska index, IM is maturity index, and IPP is phytophagous nematode index. All values are given as mean + SD; different letters represent significant differences from each other at *p* < 0.05 level.

**Table 6:** Variance analysis of the ecological index of the soil nematode community from 2018 to 2020.

Factor	S	H	C	J	R_NC_	I_W_	I_M_	I_PP_
Material	0.11	6.73 **	2.75	6.72 **	1.68	1.72	11.18 **	2.41
years	2.56	2.56	2.56	2.56	0.11	0.01	0.71	0.05
Material × Year	0.02	0.48	0.38	0.48	0.38	1.16	0.41	0.96

Note: S is species richness index, H is diversity index, C is dominance index, J is Pielou evenness index, RNC is nematode pathway index, IW is Wasilewska index, IM is maturity index, and IPP is phytophagous nematode index. ** Significant at *p*  <  0.01.

## Data Availability

The original contributions presented in this study are included in the article. Further inquiries can be directed to the corresponding author/s.
